# Health Conditions, Access to Care, Mental Health, and Wellness Behaviors in Lesbian, Gay, Bisexual, and Transgender Adults

**DOI:** 10.1155/2020/9094047

**Published:** 2020-03-05

**Authors:** Richard S. Henry, Paul B. Perrin, Ashlee Sawyer, Mickeal Pugh

**Affiliations:** Department of Psychology, Virginia Commonwealth University, Richmond, Virginia, USA

## Abstract

This study examined relationships among wellness behaviors, physical health conditions, mental health, health insurance, and access to care among a sample of 317 lesbian, gay, bisexual, and transgender (LGBT) adults. Participants completed a web-administered survey from May 2013 to April 2014. Of the sample, 41.6% of the participants reported having one or more health conditions. Most participants (92.1%) reported access to a health care facility and current health insurance coverage (84.9%), though 24.9% of those with health insurance reported being incapable of paying the copayments. Physical health conditions, age, and self-esteem explained 24% of the variance in engagement in wellness behaviors; older age, a greater number of health conditions, higher self-esteem, possession of health insurance, and ability to access to care were associated with increased wellness behaviors. Providing affordable insurance coverage, improving access to care, and properly treating mental health in LGBT individuals could improve wellness behaviors.

## 1. Introduction

On Oct. 6, 2016, the director of the U.S. National Institute on Minority Health and Health Disparities (NIMHD) announced sexual and gender minority (SGM) individuals as a designated population for health disparity research under the National Institute of Health [1]. This formal designation came about because of the increasing evidence that SGM individuals have higher burdens of certain diseases (e.g., depression, HIV/AIDs, and cancer) and less access to health care [1]. Lesbian, gay, bisexual, and transgender (LGBT) individuals were also included in the *2020 Healthy People* objectives for the first time [2]. *Healthy People* reports are 10-year U.S. national objectives for setting benchmarks and monitoring progress for improving the health of individuals and society [3]. The *2020 Healthy People* report suggests that LGBT individuals face health disparities related to discrimination, social stigma, and denial of civil and human rights [4]. This discrimination has been linked to higher rates of substance use, psychiatric disorders, and suicide [4]. These inclusions have brought greater attention to documenting and understanding the health disparities that exist in the LGBT community.

The LGBT community houses an extremely diverse set of communities, as both gender and sexual minorities are lumped under the same umbrella. These communities face many intersecting issues, including race/ethnicity, ability status, and socioeconomic issues [5]. Different segments of these communities face individual health needs and risks related to their differing social statuses. Despite these individual differences, there are common challenges to their health status [5]. Many of these challenges center on discrimination (both from providers and society), social stigma, and negative stereotypes [6]. LGBT individuals are often grouped together within the research context, despite the existence of unique subgroups; however, some group differences have been documented.

Research into sexual minorities is often limited to those with lesbian, gay, and sometimes bisexual identities. However, this work shows that sexual minorities (lesbian, gay, and bisexual individuals) as a group share increased risk for health disparities compared to heterosexual peers. LGB individuals are more likely to self-identify as having poorer health overall [5]. LBG individuals are also at higher risk for poor mental health, psychological distress, suicidal ideation, mental health disorders (e.g., depression and anxiety), disability, asthma, and physical limitations [2], as well as self-harm, and risky sexual behaviors [7]. Additionally, LGB individuals have higher rates of tobacco, alcohol, and drug use [6, 7] and experience higher rates of homelessness than heterosexual adults [7].

Lesbian and bisexual women report worse global ratings of physical health than heterosexual women [8]. They are less likely to have routine care and cancer screenings including mammography or cervical screenings [5–7]. Lesbian and bisexual women are also more likely to be overweight or obese [5, 8] and experience higher rates of asthma [2, 8] and arthritis [8]. Lesbian and bisexual women are also more likely to become disabled younger [5] and, as they age, experience higher risk of cardiovascular disease [9].

Gay and bisexual men are at increased risk for certain sexually transmitted infections (STIs) and also represent more than half of all HIV cases in the U.S. [2, 5–7]. Evidence is mixed regarding whether they experience higher risk of prostate, anal, testicular, and colon cancer [7]. Gay and bisexual men, similar to lesbian and bisexual women, are more likely to become disabled at a younger age [5]. As they age, they are also more likely to experience poor general health and to live alone [9].

There has been very limited research that has focused specifically on the health status of transgender/gender nonconforming (TGNC) individuals [2]. Trans women experience higher rates of HIV than the general population [2, 6]. Compared to the general population, TGNC individuals also experience higher rates of disability, stress, and poor mental and physical health [9]. The TGNC population experiences higher rates of suicide [7], depression, anxiety, and overall psychological distress [2]. Additionally, TGNC individuals report higher rates of military service [2], incarceration [2], victimization and sexual violence [2, 9], poor general health [2], being uninsured or underinsured [6], poverty, unemployment [7], and violent crime victimization [7].

Poor mental health has been linked to risky/negative health behaviors [10]. In one U.S.-based study examining 28 health risks, 18 were found to differ across genders and 23 were found to differ across sexual orientation [11]. Groups in this study at higher risk were transgender men and pansexual participants (self-harm), bisexual participants (substance use), and transgender women (diet and exercise behaviors) [11]. Subgroups, particularly transgender individuals and queer-identified sexual minority individuals, have limited access to care, are less likely to utilize care, and are more likely to report experience with discrimination in health care [12].

It has been well-documented that LGBT individuals have certain poorer health behaviors compared to heterosexual/cisgender peers (e.g., lesbian and bisexual women are more likely to smoke) and worse access to care [13–15]. Research into men who have sex with men likewise has documented risky health behaviors (e.g., smoking and not being HIV-tested) and having more restricted access to care [16, 17]. TGNC individuals also face challenges accessing medically necessary and culturally sensitive care, and some rely on two sets of providers, one to assist with general health care and the other to address gender-related concerns [18, 19]. This can be a financial burden, may lead to duplication in medical tests, getting differing advice on treatment, and could lead to medical errors. However, these studies have primarily documented health behaviors and access to care based on LGBT identity, without examining the relationships existing between access to care and engagement in wellness behaviors.

Broadly defined, health behaviors are those actions taken to improve or maintain health [20, 21]. Wellness behaviors, more specifically, are those behaviors which are designed not merely to prevent illness but to improve overall health and wellness [21, 22]. Wellness behaviors include things such as exercising, having regular doctor and dental visits, and gathering information about one's health [21, 22]. Being diagnosed with a chronic health condition [23] and having high self-esteem [24] are both linked with higher engagement in wellness behaviors.

The purpose of the current study was to document rates of common, potentially serious, and/or chronic health conditions, of health care insurance, and of access to care among a sample of LGBT adults. The study also sought to examine the relationships among common, potentially serious, and/or chronic health conditions, mental health, insurance status, access to care, and wellness behaviors.

## 2. Methods

### 2.1. Procedure

This study was conducted using a web-administered survey with data collection occurring from May 2013 to April 2014. Participants were recruited via online groups and forums hosted in the United States. If groups and forums were open, study information and recruitment details were posted directly to community message boards. For closed groups, moderators were contacted to post study information. Both regional and national LGBT organizations were also emailed information about the study and details about recruitment. A research coordinator screened those interested in the study to determine eligibility. Those who met the study criteria were emailed a study survey link using Research Electronic Data Capture (REDCap) a secure web-based database [25, 26] and code. Additionally, in the survey, participants were asked demographic questions including their age and to self-identify (through selecting among a list of options) an identity. Only those who selected sexual and/or gender minority options and were aged 18 or older were included in the current study.

For the study, participant inclusion criteria were to be at least 18 years old and self-identify as a sexual or gender minority. A $15 electronic http://Amazon.com gift card was provided as compensation to participants for completing the survey. An informed consent was obtained from participants, and the study was approved by the host university Institutional Review Board.

### 2.2. Participants

The sample included 317 individuals who identified as a gender or sexual minority (or both) and were at least 18 years of age. The mean age of participants was 31.0 (SD = 11.16), and the range was 18-66. Participant sexual orientation, gender identity, relationship status, employment status, and family socioeconomic status (SES) can be found in Table 1.

### 2.3. Measures

#### 2.3.1. Demographics

Survey respondents were asked to report their age (in years), gender, sexual orientation, race/ethnicity, relationship status, education, employment status, and family income level (ranges of USD).

#### 2.3.2. Health-Protective Behavior Scale (HPBS)

Health behaviors were measured using the HPBS [21, 27]. There are three subscales of the HPBS: one focused on preventative behaviors, a second on risk-taking behaviors, and a third assessing traffic risk [21]. Only the preventative behavior subscale was used in the current study, which contains 10 items answered on a five-point self-report scale ranging from 1 (*Not at all like me*) to 5 (*Very much like me*). Scores are totaled and range from 10 to 50, with higher scores indicating stronger engagement in wellness behaviors. In the current study, the internal validity was acceptable (*α* = .79).

#### 2.3.3. Hopkins Symptom Checklist 25 (HSCL-25)

Anxiety and depressive symptomology was assessed using the HSCL-25 [28]. The HSCL-25 contains a 15-item self-report measure for depression and a 10-item self-report measure for anxiety to evaluate how much over the previous week an individual was bothered or distressed by their symptoms. A mean score is calculated from participants' responses using a four-point Likert-type scale ranging from 1 (*Not at all*) to 4 (*Extremely*), with more severe symptoms indicated by higher scores. Established clinically significant cutoffs of 1.75 are used for each subscale [29, 30]. In the current study, internal validity for the anxiety items was good (*α* = .89) and excellent for the depression items (*α* = .93).

#### 2.3.4. Rosenberg Self-Esteem Scale (RSES)

Self-esteem was measured using the RSES [31]. It is a 10-item scale in which statements are rated on a four-point Likert-type scale from 0 (*Strongly disagree*) to 3 (*Strongly agree*). Scores can range from 0 to 30, and a sum score is calculated such that higher scores indicate higher self-esteem [31]. In the current study, internal validity was excellent (*α* = .91).

#### 2.3.5. Satisfaction with Life Scale (SWLS)

The SWLS is a measure of global subjective well-being without specific time parameters. There are five self-report items answered on a six-point Likert-type scale ranging from 1 (*Strongly disagree*) to 5 (*Strongly agree*). The five items each examine a different domain to create an overall satisfaction score, with higher scores indicating higher levels of satisfaction [32]. In the current study, internal validity was good (*α* = .88).

#### 2.3.6. Insurance and Access

Three researcher-generated questions were used to assess whether participants were insured (“Do you currently have health insurance”), if they otherwise had access to care (“If not, do you have access to a health care facility if you needed care”), or were able to utilize the health insurance (“Do you have health insurance, but find yourself incapable of paying your copayment for care”).

#### 2.3.7. Health Conditions

A researcher-generated questionnaire of common, potentially serious, and/or chronic health conditions regularly assessed for in primary care clinic intake forms was used to assess self-reported health conditions.

### 2.4. Data Analysis Plan

Skewness and kurtosis coefficients were calculated for the primary variables under scrutiny (anxiety, depression, self-esteem, satisfaction with life, and number of self-reported health conditions). In terms of the main analyses, first, the rates of health care access and health conditions in the sample were calculated. Second, descriptive statistics for the mental health variables, as well as health insurance and access, were calculated and presented. Third, a bivariate correlation table showing the relationships between all primary variables and demographics in the study was calculated. Fourth, the data were analyzed using a hierarchical multiple regression. In the first step, all demographic variables significantly associated with the outcome were entered. In the second step, access to care and health insurance were entered. In the third step, the number of health conditions was added. In the fourth step, mental health variables (anxiety, depression, satisfaction with life, and self-esteem) were added. All statistical analyses were performed using IBM SPSS 26 statistics software.

## 3. Results

### 3.1. Assumption Checks

All skewness coefficients were below a magnitude of .80, and all kurtosis coefficients were below a magnitude of .77, with the exception of self-reported health conditions, which had a skewness coefficient of 1.83 and kurtosis coefficient of 3.44, as would be expected given that over half of the sample did not report a health condition.

### 3.2. Mental and Physical Health Rates

Of the current survey respondents, 132 individuals (41.6%) reported having one or more health conditions. Of the 132 individuals, 76 reported just one condition (24.0%), 32 reported two conditions (10.1%), 15 reported three conditions (4.7%), 4 reported four conditions (1.3%), and 5 reported five conditions (1.6%). Thus, of the 132 individuals with health conditions, approximately 24 (7.6%) reported three or more health conditions. The most common health conditions reported were asthma (16.4%, *n* = 52), arthritis/gout (5%, *n* = 16), and thyroid disease (4.1%, *n* = 13). See Table 2 for the rates of health conditions.

For mental health, 13% (*n* = 42) of the survey respondents scored above the 1.75 cutoff for clinically significant depression and 9% (*n* = 29) for clinically significant anxiety (for mean scores, see Table 3). For satisfaction with life, the mean score was 19.36 (SD = 7.38). This score falls in the boundaries between slightly below average and average. For self-esteem, the mean score was 18.01 (SD = 6.17). Among the respondents, 32.50% (*n* = 103) scored below 15—which represents low self-esteem—while only 14.60% (*n* = 46) of respondents' scores fell in the high self-esteem range (25 or above). The mean score for engagement in preventive health behaviors was 32.32 (SD = 6.98), indicating an average endorsement of two-thirds of the preventative health behaviors.

In addition to health rates, participants were asked about health insurance status (i.e., whether they possessed health insurance), ability to pay their copay, and access to care (Table 3). The majority of participants (92.1%, *n* = 292) reported access to a health care facility if they needed once. Most of the respondents (84.9%, *n* = 269) also reported current health insurance coverage. However, of the 269 participants who stated they currently had health insurance, 24.9% (*n* = 67) stated that they found themselves incapable of paying the copayments.

### 3.3. Correlation Matrix

A correlation table was calculated showing the bivariate relationships among variables in the current study (Table 3). Wellness behaviors were correlated with all the four mental health indicators (depression, anxiety, self-esteem, and satisfaction with life) and number of health conditions. Wellness behaviors were also correlated with having health insurance and access to a health care facility but not with the ability to pay one's copay. Age was positively correlated with wellness behaviors, satisfaction with life, and number of health conditions and was negatively correlated with anxiety, depression, and having insurance but being unable to pay the copay. Family income was positively correlated with wellness behaviors, self-esteem, satisfaction with life, having health insurance, and access to health care and negatively with anxiety and depression. Education level was positively correlated with wellness behaviors, self-esteem, satisfaction with life, number of health conditions, and age and negatively with anxiety, depression, and having insurance but not being able to pay the copay.

### 3.4. Hierarchical Multiple Regression

In the hierarchical multiple regression predicting health behaviors (Table 4), demographic variables significantly related to wellness behaviors from the correlation matrix (age, family income, and education) were entered into the first step, health insurance status and access to health care into the second step, number of health conditions into the third step, and depression, anxiety, satisfaction with life, and self-esteem into the fourth step. The overall model was significant *F*(10, 306) = 9.85, *p* < .001. After controlling for demographic factors, insurance status, access to care, and number of physical health conditions, age (*β* = .21, *p* = .001), having health insurance (*β* = .12, *p* = .039), access to health care (*β* = .15, *p* = .007), and self-esteem (*β* = .18, *p* = .010) were unique predictors of engagement in wellness behaviors.

## 4. Discussion

This study examined relationships among physical health conditions, mental health, health insurance, and access to care among a sample of LGBT adults, with a specific focus on engagement in wellness behaviors. As noted in previous literature, the most influential method for reducing chronic illness is through altering individual health behaviors [33]. In the current study, LGBT adults engaged in wellness behaviors at average rates compared to the general population. A hierarchical multiple regression showed that after controlling for demographic factors, health insurance and access to care, and the number of health conditions, anxiety, depression, satisfaction with life, and self-esteem explained an additional 7% of the variance in engagement in wellness behaviors, with 24% explained overall. Within these models, older age, having a higher number of health conditions, greater self-esteem, and having health insurance and access to care were associated with greater wellness behavior engagement.

Over 40% of LGBT adults in this study reported having one or more health conditions, with asthma, arthritis/gout, and thyroid disease being the most commonly reported. These findings are similar to the rates of health conditions documented among LGBT individuals in other research which note higher rates of physical health issues among LGBT individuals [2]. A notable proportion of participants scored within the clinically significant ranges for depression and anxiety (13% and 9%, respectively). These findings are also in line with previous work noting elevated rates of mental health issues among LGBT individuals [2, 7].

The finding that number of health conditions was positively associated with engagement in wellness behaviors in both the correlation matrix and multiple regression supports existing literature showing that diagnosis of chronic health conditions can serve as a “teachable moment” for individuals and can result in increased engagement in wellness behaviors [23]. Individuals with health conditions may become motivated to make changes in their health behaviors to limit their experience of symptoms and slow the progression of the illness [23]. Having chronic health conditions has also been related to more frequent visits with medical providers [23], which can create more opportunities for providers to educate the individual about health behaviors and encourage them to engage in wellness behaviors [34].

Self-esteem was positively associated with engagement in wellness behaviors and—aside from age—represented the strongest relationship among all predictors, which supports previous work showing that higher self-esteem is related to positive health behaviors [24], psychosocial health [31], and well-being in health [35]. Recent work by Taylor and colleagues [36] has also investigated self-esteem as it relates to self-injury (including self-mutilation, attempted suicide, and substance abuse behaviors) among LGBT individuals and found that self-esteem negatively predicted this relationship above and beyond anxiety and depressive symptoms. The present study, in conjunction with previous work, suggests that feelings of self-worth among LGBT individuals may be a crucial factor in determining engagement in wellness and health promoting behaviors.

Health insurance and access to care were positively associated with wellness behaviors. This finding illustrates a connection between access to care and health behaviors. Although most participants reported having insurance and access to a health care facility (85% and 92%, respectively), approximately one out of every four of those with insurance indicated that they were unable to pay their copayment to receive care. This may indicate that although LGBT people in this study reportedly having access to health care facilities and insurance, a substantial proportion still experienced barriers in being able to utilize health services. This is an especially important issue when considering wellness, given that higher rates of health care visits have been associated with positive health outcomes [37, 38]. Health care visits serve as a good opportunity for providers to encourage engagement in health behaviors [34]. If LGBT individuals cannot afford to access health services, they may miss out on important opportunities for wellness behavior intervention.

Results from this study provide implications for clinical care and public policy. From a public health perspective, affordability of health care may be a common barrier to services among LGBT individuals. Study results also suggest that health behaviors may be malleable given that health insurance status, access to a health care facility, health conditions, and self-esteem uniquely predicted health behaviors. Increasing affordable insurance coverage, improving access to care, and properly treating mental health in LGBT individuals could have the effect of improving health behaviors.

For providers, because 41.6% of the sample endorsed one or more health conditions, there is a high necessity for established health care access and utilization for LGBT individuals. Factors related to mental health were found to correlate with wellness behaviors, which suggest that poorer mental health status may curtail proactive health care behaviors. This trend may result in the proliferation of comorbid health conditions which can lead to an exacerbation of health disparities observed in LGBT populations. Due to the elevated rates of mental health concerns among both the current study sample and previous literature, routine screeners by health care providers that sensitively assess mental health functioning and feelings about oneself (including self-esteem) as a part of treatment planning may be beneficial in identifying individuals who may need additional support in maintaining their wellness behaviors.

### 4.1. Limitations

The current study has several limitations and, as a result, directions for future research. Researchers recruited participants using web-based convenience sampling, which may not have generated a representative sample of the LGBT community. Although the sample was quite diverse in terms of gender and sexual minority identities, as well as race/ethnicity, these results can only be applied to individuals with access to the online forums similar to where the study recruited. The relationship among insurance status, access to health care services, and wellness behaviors may operate differently among populations with limited access to online forums and internet-based social networking. Thus, future research should recruit participants through both online and in-person domains in order to obtain a more inclusive sample. Another limitation of this study is that some of the data were collected before most of the Affordable Care Act (ACA) went into effect which occurred on January 1, 2014. Thus, the difference in insurance coverage status may have differentially affected health care behaviors for a part of the sample. Because the location of participants is unknown, the rollout of ACA-mandated health coverage may or may not have occurred in their state during the time of data collection. Finally, during data collection, gay and lesbian identities were collected in one category (“gay/lesbian”), and these differences cannot be distinguished, particularly for the TGNC subsample. As a result, differences in the primary variables could not be broken down by sexual orientation, and future research should consider doing so.

## 5. Conclusion

This is the first study to assess the relationships among health condition status, insurance coverage, access to care, mental health, and health behaviors in LGBT individuals. These findings underscore the moderate to high rates of physical and mental health conditions in this population and emphasize the necessity of accessible, affordable, and culturally sensitive care.

## Figures and Tables

**Table 1:** Participant demographics.

Variable	Total sample *n*	%
Age M (SD); range	31.0 (11.16); 18-66	—
Gender		
Man	89	28.1
Woman	150	47.3
Transman	26	8.2
Transwoman	29	9.1
Other	23	7.3
Sexual orientation		
Heterosexual	12	3.8
Bisexual	84	26.5
Gay/lesbian	122	38.5
Queer	80	25.2
Other	19	6
^†^Race/ethnicity		
Asian/Asian-American/Pacific islander	57	18
Black/African-American (non-Latino)	66	20.8
Latino/Hispanic	26	8.2
American-Indian/Native-American	9	2.8
White/European-American (non-Latino)	117	36.9
Multiracial/multiethnic	38	12
Other	4	1.3
Relationship status		
Long term (>12 months) with 1 person	126	39.7
New relationship (<12 months) with 1 person	39	12.3
Dating/in a relationship 1+ person	43	13.6
Not currently dating or in a relationship	109	34.4
Employment status		
Full-time	141	44.5
Part-time	47	14.8
Full-time student	41	12.9
Student and employed	53	16.7
Unemployed	35	11
Family income		
$7,000-14,999	38	12
$15,000-29,999	45	14.2
$30,000-59,999	113	35.6
$60,000-199,999	114	36
$200,000+	7	2.2
Health insurance (yes)	269	84.9
Access to a health care facility (yes)	292	92.1
Insurance, but incapable of paying copayment (yes)	79	24.9

Note. *N* = 317. ^†^Participants responded to the question “Which racial/ethnic label best describes you?” and were able to select the single best answer choice.

**Table 2:** Health conditions.

Condition	*n*	%
AIDS/HIV	4	1.3
Arthritis/gout	16	5
Asthma	52	16.4
Blood disease	2	.6
Cancer	9	2.8
Diabetes	9	2.8
Epilepsy/seizures	5	1.6
Hepatitis A	1	.3
Hepatitis B or C	4	1.3
Leukemia	1	.3
Sickle cell disease	1	.3
Thyroid disease	13	4.1

Note. *N* = 317.

**Table 3:** Correlations and means.

Variable	1	2	3	4	5	6	7	8	9	10	11	Mean	SD
1. Wellness behaviors												32.32	6.98
2. Anxiety	-.16^∗∗^											.84	.63
3. Depression	-.25^∗∗^	.76^∗∗^										.97	.7
4. Self-esteem	.27^∗∗^	-.40^∗∗^	-.58^∗∗^									18.01	6.17
5. Satisfaction with life	.34^∗∗^	-.45^∗∗^	-.60^∗∗^	.62^∗∗^								19.36	7.38
6. Health insurance? (yes)	.23^∗∗^	.02	.04	.06	.10							—	—
7. Access to a health care facility? (yes)	.23^∗∗^	-.08	-.04	.13^∗^	.05	—						—	—
8. Insurance but incapable of paying copayment? (yes)	-.02	.23^∗∗^	.18^∗∗^	-.06	-.08	—	—					—	—
9. Health condition (number)	.04	.08	-.01	-.10	.02	.09	.03	-.11				—	—
10. Age	.27^∗∗^	-.19^∗∗^	-.14^∗^	.03	.19^∗∗^	.07	.11	-.13^∗^	.38^∗∗^			—	—
11. Family income	.19^∗∗^	-.18^∗∗^	-.16^∗∗^	.24^∗∗^	.18^∗∗^	.15^∗∗^	.32^∗∗^	-.07	.00	-.01		—	—
12. Education	.25^∗∗^	-.21^∗∗^	-.19^∗∗^	.22^∗∗^	.24^∗∗^	-.01	.11^∗^	-.13^∗^	.16^∗∗^	.37^∗∗^	-.23^∗∗^	—	—

Note. *N* = 317; ^∗^*p* < .05; ^∗∗^*p* < .01. Correlations between two dichotomous variables were calculated as a Pearson correlation and between a dichotomous and continuous variable as a point-biserial correlation. Correlations between two dichotomous variables are not reported.

**Table 4:** Hierarchical multiple regression analysis: wellness behaviors.

Independent variable	Δ*R*^2^	*β*
Step 1	.12^∗∗∗^	
Age		.22^∗∗∗^
Family income		.16^∗∗^
Education		.13^∗^
Step 2	.05^∗∗∗^	
Age		.19^∗∗^
Family income		.10
Education		.15^∗^
Access to care		.11
Health insurance		.16^∗∗^
Step 3	.01	
Age		.22^∗∗∗^
Family income		.10
Education		.15^∗∗^
Access to care		.10
Health insurance		.17^∗∗^
Health conditions		−.08
Step 4	.07^∗∗∗^	
Age		.21^∗∗^
Family income		.06
Education		.11
Access to care		.12^∗^
Health insurance		.15^∗∗^
Health conditions		−.08
Anxiety		.15
Depression		−.17
Satisfaction with life		.05
Self-esteem		.18^∗^
Total *R*^2^	.24^∗∗∗^	

Note. *N* = 317; ^∗^*p* < .05, ^∗∗^*p* < .01, ^∗∗∗^*p* < .001, two-tailed.

## Data Availability

Readers can access the data supporting the conclusions of the study by contacting the corresponding author.
